# Acoustic radiation force impulse-induced lung hemorrhage: investigating the relationship with peak rarefactional pressure amplitude and mechanical index in rabbits

**DOI:** 10.1007/s10396-023-01285-z

**Published:** 2023-02-11

**Authors:** Noriya Takayama, Hideki Sasanuma, Kazuma Rifu, Naotaka Nitta, Iwaki Akiyama, Nobuyuki Taniguchi

**Affiliations:** 1 Department of Clinical Laboratory Medicine, Jichi Medical University, 3311-1 Yakushiji, Shimotsuke, Tochigi 329-0498 Japan; 2 Division of Gastroenterological, General and Transplant Surgery, Department of Surgery, Jichi Medical University, Shimotsuke, Tochigi Japan; 3 Health and Medical Research Institute, National Institute of Advanced Industrial Science and Technology (AIST), Tsukuba, Ibaraki Japan; 4 Medical Ultrasound Research Center, Doshisha University, Kyotanabe, Kyoto Japan

**Keywords:** Acoustic radiation force impulse, Elastography, Lung, Hemorrhage, Mechanical index

## Abstract

**Purpose:**

The safety of acoustic radiation force impulse (ARFI) elastography, which applies higher acoustic power with a longer pulse duration (PD) than conventional diagnostic ultrasound, is yet to be verified. We assessed the ARFI-induced lung injury risk and its relationship with peak rarefactional pressure amplitude (PRPA) and mechanical index (MI).

**Methods:**

Eighteen and two rabbits were included in the ARFI (0.3-ms push pulses) and sham groups, respectively. A 5.2-MHz linear probe was applied to the subcostal area and aimed at both lungs through the liver for 30 ARFI emissions. The derated PRPA varied among the six ARFI groups—0.80 MPa, 1.13 MPa, 1.33 MPa, 1.70 MPa, 1.91 MPa, and 2.00 MPa, respectively.

**Results:**

The occurrence of lung hemorrhage and the mean lesion area among all samples in the seven groups were 0/6, 0/6, 1/6 (1.7 mm^2^), 4/6 (8.0 mm^2^), 4/6 (11.2 mm^2^), 5/6 (23.8 mm^2^), and 0/4 (sham), respectively. Logistic regression analysis showed that derated PRPA was significantly associated with lung injury occurrence (odds ratio: 207, *p* < 0.01), with the threshold estimated to be 1.1 MPa (MI, 0.5). Spearman’s rank correlation showed a positive correlation between derated PRPA and lesion area (*r* = 0.671, *p* < 0.01).

**Conclusion:**

This study demonstrated that the occurrence and severity of ARFI-induced lung hemorrhage increased with a rise in PRPA under clinical conditions in rabbits. This indicates a potential risk of lung injury due to ARFI elastography, especially when ARFI is unintentionally directed to the lungs during liver, heart, or breast examinations.

## Introduction

Harmful side effects owing to diagnostic ultrasound modalities have not yet been reported in humans; however, some tissue damage has been reported in animal studies in organs containing gas, such as the lungs or intestines [1]. In particular, lung injury induced by pulsed ultrasound at the diagnostic modality level has been reported in previous animal experiments investigating the peak rarefactional pressure amplitude (PRPA) threshold to induce lung hemorrhage [1]. Reportedly, the B-mode or Doppler mode of clinically used diagnostic ultrasound systems induced lung hemorrhage in rats [2]. Moreover, most of the previous animal experiments inducing lung hemorrhage with diagnostic ultrasound systems were performed with 3–5 min of exposure of a fixed area of lung surface [1]. Currently, diagnostic ultrasound should be performed carefully to avoid prolonged exposure of the same organ area and minimize the time required for diagnosis, particularly for the lung, because lung hemorrhage occurrence has been reported to be dependent on exposure time as well as PRPA [3].

However, acoustic radiation force impulse (ARFI) elastography, a new diagnostic ultrasound modality, has been recently introduced in clinical practice. This relatively new technique applies ARFI “push pulses” to evaluate the stiffness of human tissue, and its use has become widespread for evaluation of liver cirrhosis or breast tumor. Push pulses used in ARFI elastography are quite different from those in conventional diagnostic ultrasound such as B-mode or Doppler mode with respect to longer pulse duration (PD) [4], which may lead to a higher risk of organ damage.

However, the safety of ARFI elastography in humans is yet to be completely guaranteed. We previously reported ARFI-induced lung hemorrhage for the first time by conducting an animal experiment with a derated PRPA of 1.3 MPa (mechanical index [MI], 0.8) using an ARFI generation system equipped with a focused 2.5-MHz probe that emitted 30 push pulses with a PD of 10 ms [5], which is a longer PD than that typically used in ARFI elastography. A subsequent experiment utilizing clinically used ARFI elastography that emitted ultrasound-containing shear wave imaging pulses and B-mode pulses other than push pulses (four 154-µs pulses per second) reportedly induced lung hemorrhage in rats, and the derated PRPA threshold for 30-s exposure was estimated to be 1.7 MPa (MI, 0.76) [6]. However, there are no previous studies on lung injury induced by ARFI that report the association with pure push pulses and PRPA.

The purpose of this study was to assess the lung injury risk associated with ARFI elastography. We hypothesized that the occurrence and severity of ARFI-induced lung injury would increase as PRPA rose, and that the threshold of MI to induce lung injury may be lower than the value currently applied in ARFI elastography under the United States Food and Drug Administration (FDA) regulations. Therefore, it is important to investigate the relationship between PRPA and lung hemorrhage for the safe use of ARFI elastography. Here, we developed an ARFI exposure model, based on our previous animal experiment, equivalent to that of ARFI elastography used in a clinical setting, and evaluated the relationship between PRPA and lung hemorrhage induced by ARFI.

## Materials and methods

### Subjects

A total of 20 male Japanese white rabbits (16–18 weeks old and weighing an average of 3.0 ± 0.2 kg) were included in this study. We conducted a series of experiments to investigate the safe use of ARFI elastography and joint research on the effect of ARFI on the heart. The materials and methods used here were partially shared between experiments. Specifically, we performed two experiments using the same rabbits on the same occasion in order to reduce the number of animals needed. Therefore, anesthesia management and ARFI systems were mutual, and this experiment on the lungs was followed by the other experiments wherein the rabbits’ hearts were exposed to ARFI to confirm that arrhythmias were evoked. The details of the heart experiments are described separately [7]. The local Institutional Care and Animal Use Committee approved the research protocol [approval number: 17233-01].

### ARFI system

**Fig. 1:**
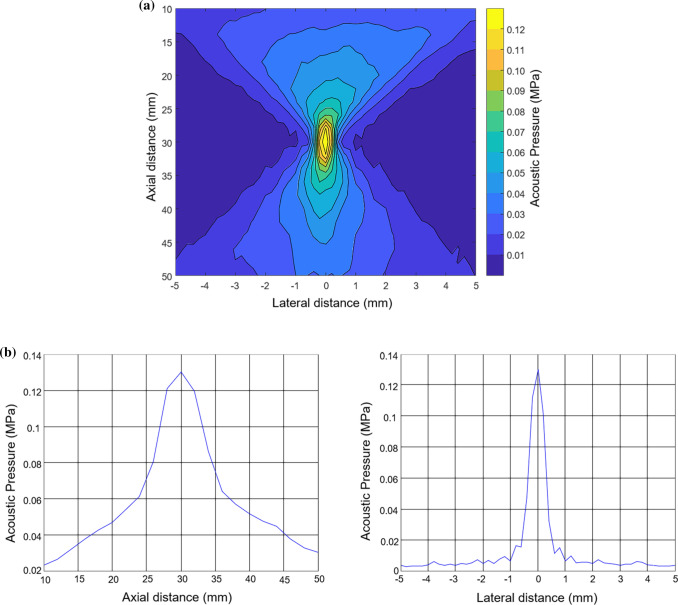
**a** Sound pressure distribution with a focal length of 30 mm generated by this system shown as an acoustic pressure contour map. Note that this map was measured under the lower sound pressure field to protect the hydrophone from damage. *ARFI* acoustic radiation force impulse. **b** Sound pressure distribution at the axial distance and at the lateral distance (axial beam width, 5–7 mm; lateral beam width, 0.5 mm; full width at half maximum, respectively)

**Fig. 2:**
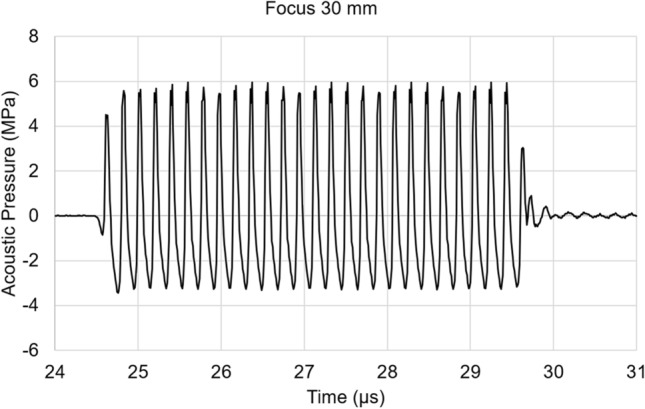
A representative acoustic pressure waveform at a focal length of 30 mm was derived from this ARFI generation system, measured in degassed water using a hydrophone. Note that the hydrophone was placed at the peak point of the sound pressure distribution in Fig. 1, where a higher input voltage was applied to generate the ARFI equivalent to this experiment

We used an ARFI generation system (Front-end Technology, Nagano, Japan) with B-mode imaging. Push pulses were emitted from a 5.2-MHz custom-made focused linear probe (pitch, 0.2 mm; 128 elements with no apodization; width, 6 mm; linear array; bandwidth, 4–15 MHz). The controller (Controller RSYS-0004; Microsonic, Tokyo, Japan) and power amplifier (Array Transmitter SYS-0013; Microsonic) were applied to generate ARFI. The acoustic pressure profile of ARFI (axial beam width, 5–7 mm; lateral beam width, 0.5 mm; full width at half maximum, respectively) generated by this system was measured in degassed water using a needle-type hydrophone (HNR-0500; Onda Corporation, Sunnyvale, CA, USA). The sound pressure distribution with a focal length of 30 mm generated by this system is shown in Fig. 1. This map was measured under the lower sound pressure field to prevent the hydrophone from damage due to continuous exposure of high acoustic pressure in spatially scanning the hydrophone. The hydrophone was then placed at the peak point of this sound pressure distribution, where a higher input voltage was applied to generate the ARFI equivalent to this experiment; a representative acoustic waveform was measured as shown in Fig. 2; and 96/128 elements were used for the focal length. The MI was proportional to a value of derated PRPA, as determined based on the Basic Concepts of Safety of Diagnostic Ultrasonic Equipment [8], using the following equation:

$${\text{MI}} = p_{{\text{r}}} /\sqrt {f_{{\text{c}}} } ,$$

*p*_r_ (in MPa) is derated PRPA that is the water-based peak negative acoustic pressure derated by 0.3 dB/cm-MHz at the location where the derated pulse intensity integral was considered the maximum and *f*_c_ is the center frequency (in MHz).

### Animal experiment procedure

**Fig. 3:**
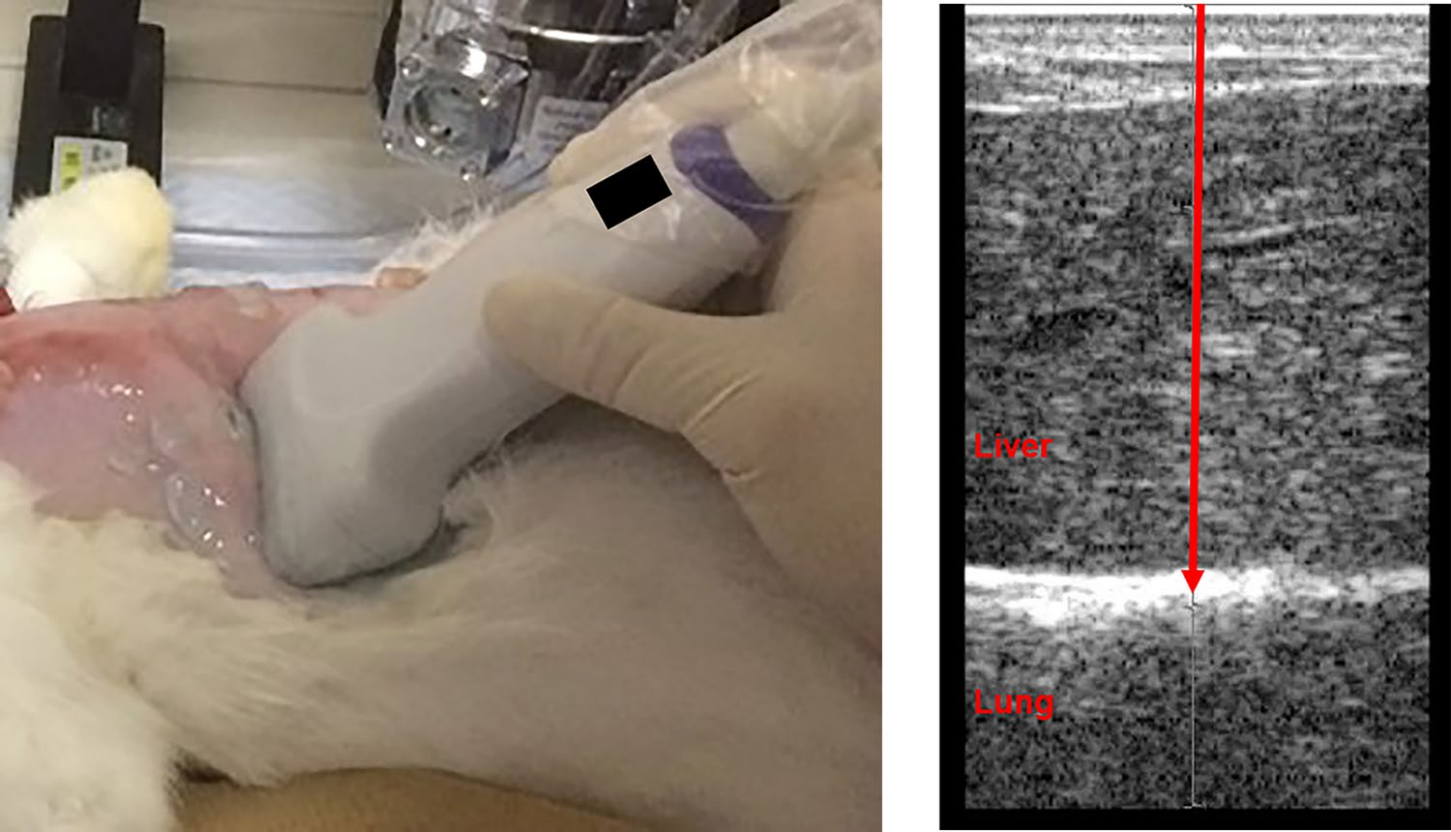
Left picture: The ARFI probe was placed on the subcostal region of the abdomen where B-mode imaging showed an appropriate location for exposure of the lung. Right picture: The red arrow shows the ARFI pathway to the lung surface through the liver at a focal point of 30 mm. *ARFI* acoustic radiation force impulse

General anesthesia was performed with an intramuscular injection of 200 µg/kg medetomidine, 0.15 mg/kg butorphanol, and 0.15 mg/kg midazolam, followed by inhalation anesthesia with sevoflurane in each rabbit at the spine position. An electrocardiogram system and pulse oximeter were attached to the rabbit to monitor the heart rate, respiratory rate, electrocardiogram, and oxygen saturation during the experiment. Tracheotomy was performed to place a tracheal tube, and manual ventilation was introduced to control breathing during the experiment. Both right and left subcostal regions were shaved and depilated, and a linear probe was placed in the region to determine the appropriate location of the lung through liver for ARFI exposure under B-mode imaging conditions, followed with an intravenous injection of 10 mg/kg propofol to stop respiration and fluctuation of the targeted lung location. Fixation of the probe was performed manually as the probe needed to be pressed into the subcostal area with subtle pressure to displace intestinal gas out of the ultrasound pathway (Fig. 3). B-mode imaging just before beginning ARFI exposure confirmed that the focal point of 30 mm was appropriately on the lung surface and that the manual fixation was stable and motionless. Subsequently, B-mode imaging was turned off to reduce its effect on the lungs, and the probe was constantly manually fixated during ARFI exposure.

Eighteen rabbits were exposed to ARFI; the study included six ARFI groups including three rabbits each. Two rabbits were not exposed to ARFI and were included in the sham group. Derated PRPA varied among the six ARFI groups: (1) 0.84 MPa (MI, 0.37), (2) 1.13 MPa (MI, 0.49), (3) 1.33 MPa (MI, 0.58), (4) 1.70 MPa (MI, 0.75), (5) 1.91 MPa (MI, 0.84), and (6) 2.00 MPa (MI, 0.88). The right and left lungs of each rabbit were exposed to ARFI. ARFI emission was first performed in one area of the right lung base followed by the left lung. The ARFI exposure settings were as follows: PD, 0.3 ms; number of push pulses, 30; and time interval between each pulse, 3 s.

After ARFI exposure, the rabbits were sacrificed under anesthesia, and thoracotomy was performed to confirm the occurrence of gross damage to the bilateral lung surface corresponding to the area of ARFI emission. Both lungs with the heart attached to the trachea were removed. The lungs were inflated using a bag valve mask through the tracheal tube inserted in the trachea for clear visualization of the lesion and for measuring the long and short diameters of the lesion at the inflated lung. Subsequently, digital photographs of the inflated lungs were taken, and image analysis software (Photoshop 2020; Adobe Inc., San José, CA, USA) was used to calculate the lesion area. Each damaged area was microscopically analyzed after fixation was initiated with formalin instillation; histologic slides were prepared with hematoxylin and eosin staining. The area including the pleura and surrounding lung tissue were observed to confirm the pathological findings of lung injury.

### Statistical analyses

All statistical analyses were performed using R software (R Foundation for Statistical Computing, Vienna, Austria; https://www.R-project.org/). Logistic regression was used to identify the dependence of incidence rates on derated PRPA at all data points for lung injury occurrence. The explanatory variable was derated PRPA; the objective variable was the probability of lesion occurrence. Logistic regression estimates were transformed to yield estimates for derated PRPA associated with 5% probabilities of lesion occurrence as the threshold. Spearman’s rank correlation was used to identify the relationship between acoustic pressure and the lesion area. The size of the lesion area was defined as zero if a lesion was not observed.

## Results

**Fig. 4:**
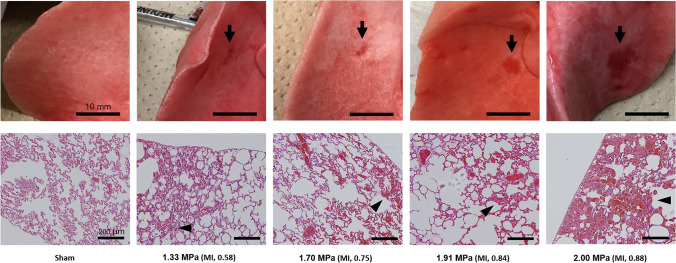
Typical lung hemorrhage induced by ARFI with a derated PRPA and MI in each group. *ARFI* acoustic radiation force impulse, *PRPA* peak rarefactional pressure amplitude, *MI* mechanical index. Upper pictures: Macroscopic analysis in groups 3–6 showed lung hemorrhage in the area (arrow) corresponding to the ARFI exposure area in each group, while there were no lesions in sham rabbits. The size of the lesion area was larger as the derated PRPA increased. Bar: 10 mm. Lower pictures: Microscopic analysis of each lesion above revealed many extravascular erythrocytes in the alveolar tissue (the cavities of the alveolar tissue indicated by the arrow head were filled with red blood cells) in groups 3–6, while erythrocytes were present only inside the vessels (the cavities of the alveolar tissue contained no red blood cells) in the sham rabbits. These histological findings in groups 3–6 are consistent with those of pulmonary alveolar hemorrhage. Bar: 200 µm

There were no significant changes in the heart rate, respiratory rate, electrocardiogram, oxygen saturation, or clinical findings related to the general appearance of the rabbits during exposure to ARFI. Hemothorax and pneumothorax were not observed in the thoracotomy findings. A lung surface lesion was observed as a red spot in the corresponding exposure area in groups 3–6 (Fig. 4). No lesions were observed in groups 1 and 2 or sham group rabbits. Microscopic findings of the red spots showed many extravascular erythrocytes in the alveolar tissue (arrow head) in groups 3–6, while erythrocytes were present only inside the vessels in the sham rabbits. These histological findings are consistent with pulmonary alveolar hemorrhaging.

The occurrence of lung injury among all samples in each group was 0/6, 0/6, 1/6, 4/6, 4/6, 5/6, and 0/4 in groups 1–6 and the sham group, respectively (Table 1). A logistic regression model showed that derated PRPA was a statistically significant factor for the occurrence of lung injury, with an odds ratio of 207 (*p* < 0.01). The coefficients of this model yielded the estimates for derated PRPA associated with 5% probabilities of lesion occurrence considering that the threshold was calculated as a derated PRPA of 1.1 MPa (MI, 0.5).

**Table 1:** Summary of lesion occurrence and mean area for derated PRPA and MI in each group

Group	Number of samples^a^	PRPA^b^ (MPa)	MI^b^	Occurrence	Mean area^c^ (mm^2^)
(1)	6	0.84	0.37	0/6	0
(2)	6	1.13	0.49	0/6	0
(3)	6	1.33	0.58	1/6	1.7 ± 1.7
(4)	6	1.70	0.75	4/6	8.0 ± 3.8
(5)	6	1.91	0.84	4/6	11.2 ± 3.7
(6)	6	2.00	0.88	5/6	23.8 ± 12.8
sham	4	0	0	0/4	0

*PRPA* peak rarefactional pressure amplitude, *MI* mechanical index

A logistic regression model showed derated PRPA was a statistically significant factor for the occurrence of lung injury, with an odds ratio of 207 (*p* < 0.01)

^a^Two samples are obtained in each rabbit exposed to acoustic radiation force impulse on right and left lungs, respectively

^b^Derated PRPA assumed to be 0.3 dB/cm-MHz deration scheme, which MI is calculated from

^c^Mean area of the lesion ± standard error

**Fig. 5:**
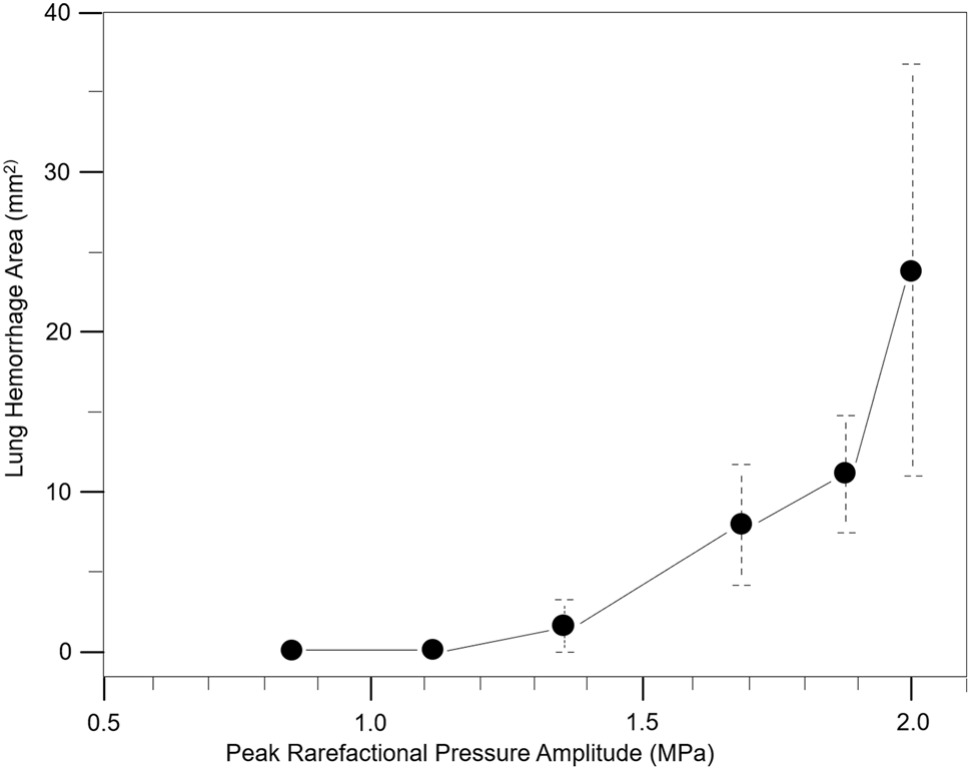
Plot of lung hemorrhage mean area with standard error bars for derated peak rarefactional pressure amplitude (PRPA) in each group. Spearman’s rank correlation showed a positive correlation between derated PRPA and the lesion area (0.671, *p* < 0.01)

The mean lesion areas in groups 3–6 were 1.7 mm^2^, 8.0 mm^2^, 11.2 mm^2^, and 23.8 mm^2^, respectively (Table 1 and Fig. 5). Spearman’s rank correlation showed a positive correlation between derated PRPA and the lesion area (*r* = 0.671, *p* < 0.01).

## Discussion

The safety of ARFI elastography is not clear even though this new technique has been widely used in clinical practice for evaluating the stiffness of human tissue. Our previous study demonstrated ARFI-induced lung injury with an MI of 0.8 in a rabbit model; however, this preliminary study contained only one case, and the ARFI settings included a PD of 10 ms, which is longer than that used in clinical practice. The current study contains the results for 20 rabbits (40 samples), including two rabbits in a sham group, with a PD of 0.3 ms, which is equivalent to the ARFI elastography used clinically. In this study, we demonstrated that our ARFI system induced lung hemorrhage, and that the occurrence and severity of ARFI-induced lung hemorrhage depended on the PRPA.

However, the mechanism of lung hemorrhage induced by ultrasound is not known yet. Some reports indicate the possibility of it resulting from cavitation as a mechanical effect [11, 12], although the possibility of a thermal effect cannot be ruled out. It is difficult to distinguish mechanical effects from thermal effects in histological findings of lung tissue. Recent reports revealed pulmonary capillary hemorrhage as a pathological finding of lung hemorrhage induced by ARFI, similar to that induced by conventional ultrasound [6, 13]. Our histology results were also consistent with the pathology. However, microscopic findings of alveolar hemorrhage generally contain less histological information. The existence of hemorrhage can be shown via the presence of extravascular erythrocytes in the alveolar tissue, but the cause often cannot be explained. Regarding the thermal effect, there is no direct evidence because it is not feasible to measure the temperature in the lungs. However, our previous report showed that the temperature increases in bone, which is rather susceptible to thermal effects—even though it was not high at a PD of 0.3 ms [14]—although a much greater thermal effect could occur in the lungs than in bone. Furthermore, the time interval of 3 s between each ARFI emission in this experiment allowed for tissue cooling, thereby prohibiting an excessive temperature rise at the ARFI exposure site. These two reasons partially support the theory that a thermal effect may be a less likely mechanism for induction of lung damage. However, the cause of ARFI-induced lung hemorrhage remains unclear. Other experiments will be needed to explore the mechanism in future studies.

Our results show that the threshold of the lung injury was estimated to be a derated PRPA of 1.1 MPa (MI, 0.5). In a previous report, the threshold of lung injury in rabbits induced by conventional diagnostic ultrasound (exposure conditions: 5.6 MHz, 10-s exposure duration, 1-kHz PRF, and 1.1-μs PD) was approximately a derated PRPA of 3.4 MPa (MI, 1.2) [9]. The results of our study indicate that ARFI can cause lung injury with a lower PRPA than that of conventional diagnostic ultrasound. The most significant difference between the two types of ultrasound is that ARFI has an approximately several hundred times longer PD than ultrasound used in conventional diagnostic modalities, while conventional diagnostic ultrasound emits several thousand times more pulses than ARFI elastography. A previous report suggested a greater likelihood of lung injury as the PD increases [10], indicating that PD is one of the significant factors inducing lung injury. Therefore, ARFI characterized by a longer PD may result in increased risk of lung injury with a lower PRPA threshold and more severe damage compared to conventional diagnostic ultrasound. Furthermore, if lung hemorrhage is induced by cavitation, which depends on the negative amplitude of the acoustic pressure, we hypothesize that phase inversion of the reflected waves at the surface of the lung would lead to greater damage because the positive amplitude of the acoustic pressure is greater than the negative amplitude in ARFI ultrasound, while positive and negative amplitudes of acoustic pressure are almost equal in conventional diagnostic ultrasound. When ARFI is emitted to lungs containing gas bodies, phase inversion occurs, and the positive acoustic wave changes to negative at the lung surface. Since the MI calculated from the inverted acoustic wave becomes larger than that of the negative amplitude of the initial acoustic wave, the MI after phase inversion at the surface of the lung could be higher. Therefore, in addition to longer PD, we hypothesize that phase inversion of ARFI could have an unexpectedly larger effect on the lungs than that of conventional diagnostic ultrasound.

To prevent cavitation-induced tissue damage during ultrasound examinations, the FDA suggests that diagnostic ultrasound modalities be performed under an MI of 1.9. This MI value is currently applied to ARFI elastography. However, our study showed that ARFI can induce lung hemorrhage with a lower PRPA than that of conventional diagnostic ultrasound. In clinical practice, ARFI elastography is used for examinations of liver, breast, or heart, which are adjacent to the lungs. Therefore, incidental ARFI exposure to the lungs is possible during examination of these organs, and the possibility of inducing lung injury in humans cannot be ruled out. All examiners who operate ARFI elastography should be aware of the possibility of this harmful side effect and avoid unintentional exposure of the lungs when the region of interest is placed near the lungs during liver, heart, or breast imaging.

### Limitations

The ARFI emission system used in this experiment was set to the assumed equivalent of push pulses generated by ARFI elastography in clinical use. However, the following two points should be considered when extrapolating these results in humans when investigating the risk of induced lung injury.

First, the ARFI system in this experiment emitted 30 push pulses per 3 s repeatedly to one area of the lung, which is a greater number of pulses than that in clinically applied ARFI elastography. Depending on the individual ARFI elastography, typically several push pulses are emitted at one tissue point during one measurement. A previous report demonstrated that shear wave elastography induced lung hemorrhage in rats even with a single frame exposure, although this mode of ARFI elastography contains four push pulses during one measurement followed by shear wave imaging pulses [6]. In the clinical practice of ARFI elastography for the evaluation of liver stiffness, 6–10 measurements are commonly performed [15]. However, every measurement with ARFI elastography is not performed at exactly the same point. In this study, 30 push pulses were emitted at one point of the lung in contrast to the clinical condition; this was regarded as the worst-case scenario. Future studies should investigate the risk of lung injury with fewer ARFI emissions, which is more equivalent to that used clinically.

Second, our findings are the results of an animal experiment using rabbits. We cannot extrapolate the risk of lung injury demonstrated in this study to humans because a larger animal tends to have a higher threshold owing to thicker pleura; the pleura thickness is considered an important factor for increasing the threshold of lung hemorrhage induced by ultrasound [1, 16]. For these reasons, the threshold for lung injury in humans induced by ARFI is higher than the result in our study of rabbits. A study using a large animal is needed to determine the safety of ARFI in clinical use and investigate the risk of lung injury in humans.

## Conclusion

This study demonstrated that the occurrence and severity of ARFI-induced lung hemorrhage increased with a rise in PRPA under clinical conditions, which was within the MI (< 1.9) mandated by the FDA. Statistical analyses revealed that the threshold to induce lung injury was estimated to be a derated PRPA of 1.1 MPa (MI, 0.5) for rabbits. This indicates a potential risk of lung injury due to ARFI elastography, especially when the ARFI is unintentionally directed to the lungs during liver, heart, or breast examinations.

## Data Availability

The data that support the findings of the study are available from the corresponding author on reasonable request.
