# Correction to: Acoustic radiation force impulse-induced lung hemorrhage: investigating the relationship with peak rarefactional pressure amplitude and mechanical index in rabbits

**DOI:** 10.1007/s10396-024-01421-3

**Published:** 2024-03-02

**Authors:** Noriya Takayama, Hideki Sasanuma, Kazuma Rifu, Naotaka Nitta, Iwaki Akiyama, Nobuyuki Taniguchi

**Affiliations:** 1 Department of Clinical Laboratory Medicine, Jichi Medical University, 3311-1 Yakushiji, Shimotsuke, Tochigi 329-0498 Japan; 2 Division of Gastroenterological, General and Transplant Surgery, Department of Surgery, Jichi Medical University, Shimotsuke, Tochigi Japan; 3 Health and Medical Research Institute, National Institute of Advanced Industrial Science and Technology (AIST), Tsukuba, Ibaraki Japan; 4 Medical Ultrasound Research Center, Doshisha University, Kyotanabe, Kyoto Japan

**Correction to: Journal of Medical Ultrasonics (2023) 50:143–150** 10.1007/s10396-023-01285-z

The article “Acoustic radiation force impulse‑induced lung hemorrhage: investigating the relationship with peak rarefactional pressure amplitude and mechanical index in rabbits”, written by Noriya Takayama, Hideki Sasanuma, Kazuma Rifu, Naotaka Nitta, Iwaki Akiyama and Nobuyuki Taniguchi, was originally published Online First without Open Access. After publication in volume 50, issue 2, page 143–150 the author decided to opt for Open Choice and to make the article an Open Access publication. Therefore, the copyright of the article has been changed to © The Author(s) 2023 and the article is forthwith distributed under the terms of the Creative Commons Attribution 4.0 International License, which permits use, sharing, adaptation, distribution and reproduction in any medium or format, as long as you give appropriate credit to the original author(s) and the source, provide a link to the Creative Commons licence, and indicate if changes were made. The images or other third party material in this article are included in the article’s Creative Commons licence, unless indicated otherwise in a credit line to the material. If material is not included in the article’s Creative Commons licence and your intended use is not permitted by statutory regulation or exceeds the permitted use, you will need to obtain permission directly from the copyright holder. To view a copy of this licence, visit http://creativecommons.org/licenses/by/4.0.

The original article has been corrected.

